# Initial Experience With Trans-anal Minimally Invasive Surgery (TAMIS) for Rectal Polyps and Early Colorectal Cancers at Cumberland Infirmary, Carlisle

**DOI:** 10.7759/cureus.31958

**Published:** 2022-11-28

**Authors:** Badreldin Mohamed, Mayat Aung, Aboalgasim Mohammed, Mohamed Mohamed, Mohamed Edilbe

**Affiliations:** 1 Vascular Surgery/General Surgery, Cumberland Infirmary, Carlisle, GBR; 2 Colorectal Surgery, Cumberland Infirmary, Carlisle, GBR; 3 Gatroentrology, Cumberland Infirmary, Carlisle, GBR; 4 Gastroenterology, Gateshead Hospital, Newcastle upon Tyne, GBR

**Keywords:** early rectal cancer, adenocarcinoma, tubulovillous adenoma, tamis, cumberland infirmary, rectal polyps

## Abstract

Introduction: Bowel cancer is the fourth most common type of cancer in the United Kingdom in 2019. Total mesorectal excision is the standard procedure for the removal of rectal tumors, however, it comes with serious side effects. Therefore, less invasive procedures and sphincter preservation techniques have been developed, like conventional trans-anal excision, and trans-anal endoscopic microsurgery (TEM). In 2010, trans-anal minimally invasive surgery (TAMIS) was introduced as an alternative to TEM, which offers the same benefits as TEM but at a lower cost and without the need for specialized instrumentation. This study aims to assess the practicability and safety of this technique and to report its findings.

Methods: Retrospective data of all patients who underwent TAMIS at Cumberland Infirmary (Carlisle, UK) from July 2017 to July 2022 for large benign rectal polyps or early rectal cancer were collected. Variables collected included patients' age, gender, number of procedures per year, perioperative outcome, and histopathology outcome. The SPSS version 21 (IBM Corp., Armonk, NY, USA) was used for both descriptive and inferential analyses of the data.

Results: During a five-year period, 42 patients underwent TAMIS at Cumberland Infirmary. The primary indication for TAMIS was distal rectal lesions, large rectal polyps up to 120 mm, and early rectal cancer (T1). The median age of the assessed patients at the time of surgery was 71 years with 64.29% (27) male and 35.71% (15) female. The mean operating time was 123 minutes (range 45 to 240 minutes). The surgical and pathological outcome included a mean polyp size of 6 cm (+/- 0.8 cm), a rate of specimen fragmentation at 19.04% (n=8), and a rate of positive margins at 04.76% (n=2), whereas histology of 73.81% (n=31) was tubulovillous adenoma and 11.91% (n=5) was adenocarcinoma. There was no 30-day postoperative mortality, however, the 30-day re-operation rate was 02.39% (n=1) and the recurrence rate which needed further intervention was 26.19% (n=11).

Conclusion: Our findings suggest that TAMIS produces positive results. The size of the lesions removed, and the effect of an early learning curve are reflected in the rate of specimen fragmentation and polyp recurrence. Nonetheless, TAMIS is a safe and effective alternative to total mesorectal excision for certain types of rectal lesions and should be used for more proximal and complex rectal lesions.

## Introduction

In 2019, bowel cancer accounted for 12% (43438 cases) of all new cancer diagnoses in the United Kingdom (UK); after breast cancer, prostate cancer, and lung cancer, it was the second leading cause of cancer death [1]. More cases of down-staged or early-stage colorectal cancer are being treated as a result of increased screening rates and the implementation of chemotherapy management protocols [2,3]. 

Total mesorectal excision is the standard of care for rectal tumors, but it comes with serious side effects like anorectal complications, a non-functioning stoma, or a permanent stoma, as well as sexual and urinary problems [2,3]. For large polyps and early tumors without lymph node invasion, local excision is considered a curative procedure, and there is a growing trend toward less invasive procedures and sphincter preservation techniques [4]. There are different approaches to local excision such as conventional trans-anal excision using open surgery instruments under direct vision, or trans-anal endoscopic microsurgery (TEM) [5].

In 1983, Dr. Gerhard Buess pioneered TEM, which employs insufflation to maintain pneumorectum and a fixed rigid recto scope to ensure a clear view [6]. Although TEM has a higher rate of negative microscopic margins (5 mm negative margin adequate for adenomas whereas 10 mm negative margin adequate for cancers), lower specimen fragmentation, and a similar recurrence rate to conventional trans-anal excision (but the same postoperative complication rate), its complexity, high cost, and short learning curve have limited its use [7-9]. Atallah et al. (2010) introduced trans-anal minimally invasive surgery (TAMIS) as an alternative to TEM, which offers the same benefits at a lower cost and without the need for specialized instrumentation as well as an easier setup and greater procedural flexibility, a shallow learning curve, and better outcomes for mid and high rectal lesions [10,11]. Although TAMIS is already commonplace in many hospitals, it wasn't until almost five years ago that our facility adopted it. The purpose of this study is to assess the practicability and safety of this technique and to report its findings.

## Materials and methods

Information was gathered retrospectively from medical records for patients of either gender who were over the age of 18 and who had undergone TAMIS at Cumberland Infirmary (Carlisle, UK) for large benign rectal polyps (up to 120 mm) or early rectal cancer (T1N0M0) between July 2017 and July 2022. 

Tumors that had been removed by means other than trans-anal sub-mucosal resection or those that had already recurred at presentation were not included. The need for patient consent and approval from the research council was discounted because the study was observational and did not involve identifying information about individual patients. Specimen loss, negative margin, recurrence rate, operative time, hospital stay, and post-procedure complications were among the data points collected. 

The SPSS version 21 (IBM Corp., Armonk, NY, USA) was used for both descriptive and inferential analyses of the data. In our institution, patients undergoing TAMIS follow a protocol that includes preoperative staging for malignant lesions via CT of the chest, abdomen, and pelvis, pelvic MRI, and discussion at the multidisciplinary team (MDT) level. After discussing the advantages and disadvantages of the TAMIS procedure with the patient, consent was obtained. The TAMIS was performed under general anesthesia by two colorectal consultants trained in the procedure. Formalin-fixed specimens were sent off to histopathology. Sigmoidoscopy (in two to six months to check TAMIS site then after a further 12 months and 18 months if there is recurrence at TAMIS site) and MRI scans are performed routinely as part of follow-up care. National guidelines for cancer patients' follow-up were observed (colonoscopy in one year and one-off surveillance colonoscopy three years later).

## Results

The study group evaluated during the specified period comprised 42 patients, of which 64% were male. The age range was between 40 and 92 years, with a median of 71 years, and up to 50% of this patient population was between the ages of 70 and 80 (Table 1). 

**Table 1:** Patients' demographics

		Number of patients	Percentage
Gender	Male	27	64.29
	Female	15	35.71
Age (years)	40-50	1	2.38
	50-60	4	9.52
	61-70	7	16.67
	71-80	21	50.00
	81-90	8	19.05
	>90	1	2.38

**Figure 1:**
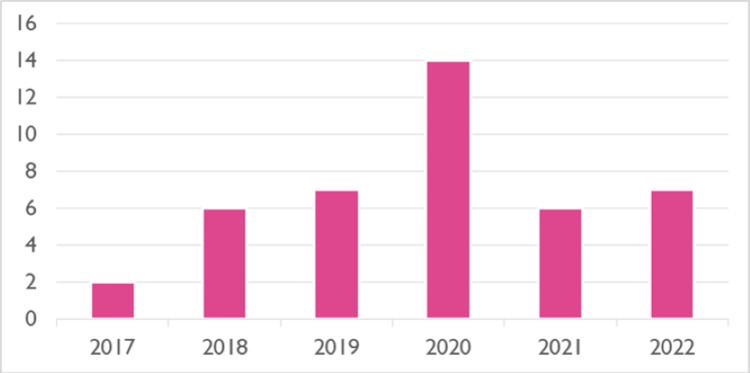
Number of TAMIS procedures performed each year (2017 to 2022) TAMIS: Trans-anal minimally invasive surgery

Our records show that since the start of TAMIS service in July 2017, the number of patients increased gradually till 2020. However, it decreased afterward due to the impact of COVID on hospital resources and list availability (Figure 1).

In our study group, the mean operative time ranged from 45 to 240 minutes, averaging 123 minutes, and there was no peritoneum breach. The majority of procedures (71%) were day cases; however, seven patients (16%) stayed overnight due to two patients developing urinary retention and five patients requiring observation and pain management. Four patients experienced pyrexia following the TAMIS procedure, with two patients admitted for two days and the remaining two patients admitted for four and five days, respectively. One patient admitted six days after TAMIS treatment required a colonoscopy and blood transfusion for bleeding (Table 2).

**Table 2:** Perioperative outcomes

Parameter		Number of patients (percentage)
Mean operative time (minutes)		123 (range: 45 to 240 minutes)
Breach of the peritoneal cavity		None
Closure of the rectal defect		6 patients (14.29%)
Incomplete excision		4 (one on excisional biopsy and three on piecemeal excisions)
Length of hospital stay	Day case	30 Patients (71.43%)
	Overnight stay	07 Patients (16.67%)
	Two days	02 Patients (04.76%)
	Four days	01 Patients (02.38%)
	Five days	01 Patients (02.38%)
	Six days	01 Patients (02.38%)

**Figure 2:**
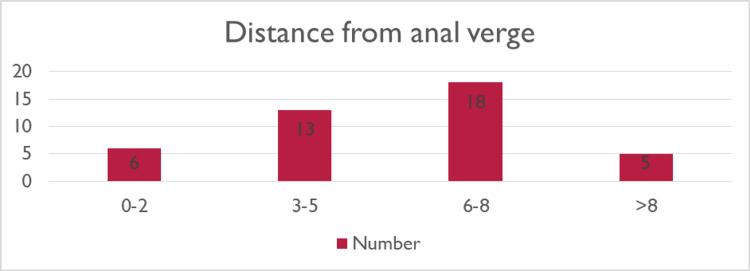
Distance from anal verge in centimeters (cm)

The median distance from the anal verge was 7.6 cm with a range of 2 cm to 11 cm (Figure 2).

**Figure 3:**
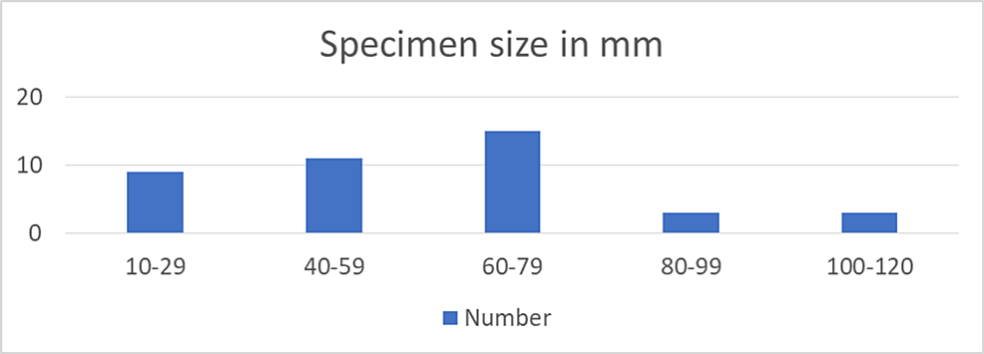
Specimen size in millimeters (mm)

Histological findings of the submitted specimen are shown in Table 3 while specimen size in millimeters (mm) is shown in (Figure 3). Five patients have adenocarcinoma on the final histology reports, none of them have a history of other malignancies and just one patient had a family history of colon cancer (father). 

**Table 3:** Histology outcome

Parameter		Number of cases (Percentage)
Mean polyp size		6cm (+/- 0.8 cm) (Range: 1 to 12cm)
Specimen fragmentation		8 (19.04%)
Positive margins		2 (04.76%)
Excision thickness	Mucosectomy	34 (80.95%)
	Partial thickness	02 (04.76%)
	Full thickness	06 (14.29%)
Final pathology	Tubulovilous adenoma	31 (73.81%)
	Tubular adenoma	04 (09.52%)
	Villous adenoma	01 (02.38%)
	Serrated adenoma	01 (02.38%)
	Adenocarcinoma	05 (11.91%)

The median duration of patient follow-up was 20 months (range: one to 45 months). Eleven patients (26.91%) had a recurrence out of which four patients (9.52%) underwent endoscopic mucosal resection (EMR) as the recurrent lesions were endoscopically suitable for EMR and the histology of the primary lesion was adenoma; two patients (4.76%) underwent extralevator abdominoperineal resection (ELAPE); and two patients had abdominal perineal resection (APR) as they had local recurrence staged asT2 N0M0. On follow-up sigmoidoscopy seven patients underwent polypectomy for initially suspected recurrence, out of which the histology of four patients was granulation tissue and the histology of the other three patients was recurrent adenomas. The APR was performed on two patients (4.76%) who did not qualify for TAMIS. One patient with refractory bleeding failed to respond to conservative measures and required colonoscopy, clips to control the bleeding, and post-procedure blood transfusion on the same admission, and his overall hospital stay was six days. In our study group, no cases of mortality or readmission were reported.

## Discussion

The TAMIS is a new procedure for the resection of rectal polyps and early rectal cancer that is a hybrid of TEM and laparoscopic surgery using conventional laparoscopic instruments, a standard laparoscopic carbon dioxide (CO2) insufflator, and cameras with a single port incision. According to the national comprehensive cancer network (NCCN), the indication for resection of rectal lesions is a mobile lesion measuring less than 3 cm, not extending beyond the sub mucosa or covering one-third of the circumference of the bowel and having moderate to good differentiation [12]. The TAMIS has many advantages over TEM, including 360 degrees vs. 220 degrees of visibility within the rectal lumen, the availability of operative instruments (laparoscopic), the ease of positioning the patient in the operative theatre, and the short setup time, which is as quick as two minutes in TAMIS compared to 30 to 45 minutes in TEM [13,14].

In a 2014 systematic review of 390 TAMIS patients, Martin-Perez et al. found that the average size of the lesions resected was 3.1 cm (range: 0.8 cm to 4.75 cm) and the mean distance from the anal verge was 7.6 cm (range: 3 cm to 15 cm), whereas in our study the average size of the lesion was 6 cm (range: 1 cm to 12 cm) and the average distance from the anal verge was 6 cm (range: 2 cm to 11 cm) [15]. The ability to perform an en-bloc resection with clear margins is cited as a justification for this approach. However, 5% of specimens showed a positive resection margin in Martin-Perez et al.'s study, and 31% were too fragmented for them to assess the resection status under the microscope [15]. In contrast, Haugvik et al. found that 22% of patients had positive-resection margins on the final pathology specimen reports, however, we found in our study only 4% of our patients had such a positive margin, and that 19% of our specimens were fragmented [16].

A series by Keller et al. reported 78.67% of the patients had benign histology; in our study, 82.09% of the cases had benign final histology [17]. In the case of malignant lesions, a full-thickness excision must be performed to achieve a negative margin of at least 1 mm [17]. In our study, the resection limits were mucosectomy in 34 patients (80.95%), partial-thickness in two patients (4.76%), and full thickness in six patients (14.29%); all of them were malignant lesions except for one patient, and there was no breach of the peritoneum reported in our group. Rectal closure was performed in six patients (14.29%) via TAMIS port. However, many authors reported peritoneal entry. Quaresma et al. reported 16.2% (5/31) of their TAMIS cases having peritoneal entry, and Caycedo et al. reported 10% (5/50) having peritoneal entry [11,18,19].

In a systematic review of 259 patients, Martin-Perez et al. found that the recurrence rate was 2.7% with a mean follow-up of 7.1 months, which is better than the reported rate of 17% for local recurrence after TEM [1,15,20]. Recurrence rates reported by Albert et al. were 4.3%, and 8% (four of 50) in the study Caycedo-Marulanda et al. [21]. In our study, we found that the recurrence rate was 26.19% with three recurrent adenomas confirmed following polypectomy in seven patients (16.67%), whereas the management of the other recurrence cases involved ELAPE resection in two patients (4.76%), EMR in four patients (9.52%), and APR in two patients (4.76%), all of whom were followed for an average of 20 months.

Minor complications have been reported in the literature. The most common is post-procedural hemorrhage, which can be treated conservatively and if it fails then the bleeding can be stopped using endoscopy or by examining the patient while they are under general anesthesia and over-sewing the incision [22-24]. In our study, only one patient (2.38%) required a colonoscopy because of bleeding that couldn't be stopped with conservative management and required a colonoscopy and clips applied to control the bleeding. Additionally, other complications include transient pyrexia (in 4% of patients), and urinary retention (in 4% of patients) [22,23]. Although the reported incidence of diverting stoma in the setting of TEM is 0% to 14%, none has been reported in our study group [20].

The limitations of our study are the relatively small number of the study group partly because of a small number of performing surgeons (two consultants), the early learning curve of the performing consultants, the impact of COVID on hospital resources, and the list availability which is noticed in the number of procedures done in 2021 and early 2022. The majority of the cases are benign lesions (88.09%). This is because of the early learning curve of the performing consultants. The study is a retrospective study that relies on other accurate records and is subject to bias.

## Conclusions

In comparison to the existing literature, our findings suggest that TAMIS produces positive results. The size of the polyps removed, and the effect of an early learning curve are reflected in the rate of specimen fragmentation and polyp recurrence in our study group. However, no disabling morbidity or mortality was reported in our institution and the majority of the assessed patients had day surgery. Therefore, TAMIS is a safe and effective alternative to major surgical procedures such as radical mesorectal excision for the management of large rectal polyps and early rectal cancers. 

## References

[1] (2022). UK Cancer Statistics and Data | World Cancer Research Fund UK. https://www.wcrf-uk.org/preventing-cancer/uk-cancer-statistics/.

[2] Stephens RJ, Thompson LC, Quirke P (2010). Impact of short-course preoperative radiotherapy for rectal cancer on patients' quality of life: data from the Medical Research Council CR07/National Cancer Institute of Canada Clinical Trials Group C016 randomized clinical trial. J Clin Oncol.

[3] Peeters KC, van de Velde CJ, Leer JW (2005). Late side effects of short-course preoperative radiotherapy combined with total mesorectal excision for rectal cancer: increased bowel dysfunction in irradiated patients—a Dutch colorectal cancer group study. J Clin Oncol.

[4] Rullier E, Vendrely V, Asselineau J (2020). Organ preservation with chemoradiotherapy plus local excision for rectal cancer: 5-year results of the GRECCAR 2 randomised trial. Lancet Gastroenterol Hepatol.

[5] Paty PB, Nash GM, Baron P (2002). Long-term results of local excision for rectal cancer. Ann Surg.

[6] Buess G, Theiss R, Günther M, Hutterer F, Pichlmaier H (1985). Endoscopic surgery in the rectum. Endoscopy.

[7] Clancy C, Burke JP, Albert MR, O'Connell PR, Winter DC (2015). Transanal endoscopic microsurgery versus standard transanal excision for the removal of rectal neoplasms: a systematic review and meta-analysis. Dis Colon Rectum.

[8] de Graaf EJ, Burger JW, van Ijsseldijk AL, Tetteroo GW, Dawson I, Hop WC (2011). Transanal endoscopic microsurgery is superior to transanal excision of rectal adenomas. Colorectal Dis.

[9] Moore JS, Cataldo PA, Osler T, Hyman NH (2008). Transanal endoscopic microsurgery is more effective than traditional transanal excision for resection of rectal masses. Dis Colon Rectum.

[10] Melin AA, Kalaskar S, Taylor L, Thompson JS, Ternent C, Langenfeld SJ (2016). Transanal endoscopic microsurgery and transanal minimally invasive surgery: is one technique superior?. Am J Surg.

[11] Caycedo-Marulanda A, Jiang HY, Kohtakangas EL (2017). Transanal minimally invasive surgery for benign large rectal polyps and early malignant rectal cancers: experience and outcomes from the first Canadian centre to adopt the technique. Can J Surg.

[12] Heafner TA, Glasgow SC (2014). A critical review of the role of local excision in the treatment of early (T1 and T2) rectal tumors. J Gastrointest Oncol.

[13] Atallah S, Albert M, Larach S (2010). Transanal minimally invasive surgery: a giant leap forward. Surg Endosc.

[14] Karakayali FY, Tezcaner T, Moray G (2015). Anorectal function and outcomes after transanal minimally invasive surgery for rectal tumors. J Minim Access Surg.

[15] Martin-Perez B, Andrade-Ribeiro GD, Hunter L, Atallah S (2014). A systematic review of transanal minimally invasive surgery (TAMIS) from 2010 to 2013. Tech Coloproctol.

[16] Haugvik SP, Groven S, Bondi J, Vågan T, Brynhildsvoll SO, Olsen OC (2016). A critical appraisal of transanal minimally invasive surgery (TAMIS) in the treatment of rectal adenoma: a 4-year experience with 51 cases. Scand J Gastroenterol.

[17] Keller DS, Tahilramani RN, Flores-Gonzalez JR, Mahmood A, Haas EM (2016). Transanal minimally invasive surgery: review of indications and outcomes from 75 consecutive patients. J Am Coll Surg.

[18] Shimada Y, Takii Y, Maruyama S, Ohta T (2011). Intramural and mesorectal distal spread detected by whole-mount sections in the determination of optimal distal resection margin in patients undergoing surgery for rectosigmoid or rectal cancer without preoperative therapy. Dis Colon Rectum.

[19] Quaresima S, Balla A, Franceschilli L (2016). Transanal minimally invasive surgery for rectal lesions. JSLS.

[20] Albert MR, Atallah SB, deBeche-Adams TC, Izfar S, Larach SW (2013). Transanal minimally invasive surgery (TAMIS) for local excision of benign neoplasms and early-stage rectal cancer: efficacy and outcomes in the first 50 patients. Dis Colon Rectum.

[21] Wexner SD, Berho M (2014). Transanal TAMIS total mesorectal excision (TME)-a work in progress. Tech Coloproctol.

[22] Gorgun IE, Aytac E, Costedio MM, Erem HH, Valente MA, Stocchi L (2014). Transanal endoscopic surgery using a single access port: a practical tool in the surgeon's toybox. Surg Endosc.

[23] Hompes R, Ris F, Cunningham C, Mortensen NJ, Cahill RA (2012). Transanal glove port is a safe and cost-effective alternative for transanal endoscopic microsurgery. Br J Surg.

[24] Marks JH, Frenkel JL, Greenleaf CE, D'Andrea AP (2014). Transanal endoscopic microsurgery with entrance into the peritoneal cavity: is it safe?. Dis Colon Rectum.

